# Commentary: Fighting Depression: Action Video Game Play May Reduce Rumination and Increase Subjective and Objective Cognition in Depressed Patients

**DOI:** 10.3389/fpsyg.2018.01844

**Published:** 2018-10-01

**Authors:** Arnav Gupta, Veeral Desai, Michael Wong

**Affiliations:** ^1^Bachelor of Health Sciences Program, McMaster University, Hamilton, ON, Canada; ^2^Psychology Department, University of Wisconsin - La Crosse, La Crosse, WI, United States

**Keywords:** video games, rumination, cognition, depression, attentional control, executive functioning

## Introduction

In recent years, there has been growing interest in the potential of video games to improve health and cognition (Russoniello et al., 2013; Allen et al., 2017; Bediou et al., 2018; Desai et al., 2018). In “Fighting Depression: Action Video Game Play May Reduce Rumination and Increase Subjective and Objective Cognition in Depressed Patients,” Kühn et al. (2018) investigated the potential effect of an action-based video game, Boxon X, on rumination, depression, and cognition. To do this, Kühn et al. (2018) recruited 68 patients who were clinically diagnosed with depression for their study. The patients were randomized into one of two groups: a training group, who played Boson X, and a waitlist control group. The results revealed no beneficial effect of playing Boson X on depression and cognition, and a (non-statistically significant) trend for rumination to decrease.

Rumination, traditionally associated with poor attentional control, has been suggested to lead to difficulty disengaging from negative thoughts; thus, rumination has been implicated in depression (Hsu et al., 2015). This rumination-depression relationship has led some interventions to target rumination as a means of reducing depressive symptoms. For example, Watkins et al. (2011) found that participants with depression and who received cognitive behavior therapy that targeted (and reduced) rumination saw greater alleviation of depressive symptoms than those in a control group. We speculate here that the gameplay mechanics of Boson X may not sufficiently target attentional control (and related cognitive abilities), and thus rumination and depression. Recognizing the diversity of video games, we hope, in this commentary, to discuss how gameplay duration and video game genre may impact depression and cognition.

## Impact of gameplay duration

One factor that may limit the video games' effectiveness in reducing rumination is a temporal threshold for sufficient cognitive activation. In a meta-analysis, Bediou et al. (2018) observed a medium effect size (Hedge's g = 0.55) between habitual action video game playing and enhanced cognition. On a cortical level, Benady-Chorney et al. (2018) observed that habitual video game players (who played ≥ 6 h/week for a 6 month period) compared to non-habitual video game players (who played ≤ 6 h/week for a 6 month period) had greater cortical thickness in the dorsal anterior cingulate cortex, which is associated with enhanced learning and attentional control. The lack of a positive result in Kühn et al. (2018) perhaps suggests the gameplay duration (6 weeks vs. 6 months in Benady-Chorney et al., 2018) may not have induced sufficient cortical activation for cognitive enhancements and subsequent reduction of rumination (and depressive symptoms).

## Impact of genre

Another possibility for the lack of a positive result is perhaps explained by the impact of video game genre, as studies have suggested different video game genres have differential effects on cognitive abilities. Indeed, a meta-analysis by Powers et al. (2013) on the effects of video game play on information processing highlighted how the genre of the video game can influence how effective the video game is at enhancing aspects of cognitive functioning (e.g., auditory and visual processing, executive functions, and spatial imagery). Kühn et al. (2018) used an action video game, a genre that Powers et al. (2013) reported had a moderate effect size (Cohen's d = 0.22–0.62) with respect to cognitive functioning. Research has shown that general cognitive activation and executive functioning are more linked to games that involved learning patterns and logical puzzles, components not found in Boson X, than those that are classified as action video games (Mondéjar et al., 2016). Subsequently, Boson X may not be an effective medium for the alleviation of depressive symptoms perhaps because it does not target the cognitive abilities that have been implicated in rumination (and depression). That said, it is interesting that some studies have found that playing action video games reduces depressive symptoms (Ferguson and Rueda, 2010). This raises the question: Why are some action video games effective at reducing depression and others not?

Dobrowolski et al. (2015) discussed how studies often label “action video games” as a broad category, grouping games that contain various gameplay mechanics (actions required for in-game progression). The authors expand on how these studies often fail to control for the variability that arises from differences in gameplay mechanics, suggesting that the cognitive effects of playing video games may differ even within a genre. This is supported by Mondéjar et al. (2016) who found that variations in gameplay mechanics of video games within the same genre can activate different parts of the brain. The authors classified games into five key genres, notably subdividing action video games into two categories: timely action and accurate action. *Timely action* games prioritize the precise timing of an action, whereas *accurate action* games prioritize the precise execution of an action. Given these definitions, it seems Boson X is most accurately classified as a timely action game, since it places emphasis on the precise timing of a series of jumps. According to Mondéjar et al. (2016), timely, unlike accurate, action games do not target cognitive abilities such as attention and impulse control that have been associated with rumination (Hsu et al., 2015); this perhaps explains the lack of a statistically significant decrease in rumination in Kühn et al. (2018).

Furthermore, Bernstein et al. (2017) recently suggested the traditional view of rumination as a single construct may be too simplistic; using network analysis, the authors showed that rumination encompasses multiple components, including, for example, perseveration, brooding, and self-criticism. Although rumination has been linked to depression, this finding suggests the rumination-depression link may be more complicated than traditionally thought, especially if certain components of rumination (e.g., self-criticism) are more linked to depression than others. This further stresses the importance of tailoring gameplay mechanics to the components/conditions of interest in video game interventions.

## Conclusion

Kühn et al. (2018) did not report any obvious reduction in rumination (and alleviation of depressive symptoms) among their participant sample. This may be explained by Boson X's inability to appropriately target cognitive processes that have been implicated in rumination (e.g., attentional control). Given current literature on the differential effects of gameplay duration and mechanics, future studies should investigate how these factors may influence symptom alleviation and provide insight into the viability of video game-based treatments for the condition of interest (e.g., depression). Additionally, it would be important in video game research to classify video games based on the cognitive processes that are involved.

## Author contributions

All authors listed have made a substantial, direct and intellectual contribution to the work, and approved it for publication.

### Conflict of interest statement

The authors declare that the research was conducted in the absence of any commercial or financial relationships that could be construed as a potential conflict of interest.
